# Multi-Objective Optimization for Resource Allocation in Space–Air–Ground Network with Diverse IoT Devices †

**DOI:** 10.3390/s25010274

**Published:** 2025-01-06

**Authors:** Yongnan Xu, Xiangrong Tang, Linyu Huang, Hamid Ullah, Qian Ning

**Affiliations:** College of Electronics and Information Engineering, Sichuan University, Chengdu 610065, China; yongnanxu@stu.scu.edu.cn (Y.X.); xiangrongtang@stu.scu.edu.cn (X.T.); lyhuang@scu.edu.cn (L.H.); hamidullah@stu.scu.edu.cn (H.U.)

**Keywords:** UAV, internet of things, system capacity, space–air–ground integrated network, multi-objective optimization evolutionary algorithm

## Abstract

As the Internet of Things (IoT) expands globally, the challenge of signal transmission in remote regions without traditional communication infrastructure becomes prominent. An effective solution involves integrating aerial, terrestrial, and space components to form a Space–Air–Ground Integrated Network (SAGIN). This paper discusses an uplink signal scenario in which various types of data collection sensors as IoT devices use Unmanned Aerial Vehicles (UAVs) as relays to forward signals to low-Earth-orbit satellites. Considering the fairness of resource allocation among IoT devices of the same category, our goal is to maximize the minimum uplink channel capacity for each category of IoT devices, which is a multi-objective optimization problem. Specifically, the variables include the deployment locations of UAVs, bandwidth allocation ratios, and the association between UAVs and IoT devices. To address this problem, we propose a multi-objective evolutionary algorithm that ensures fair resource distribution among multiple parties. The algorithm is validated in eight different scenario settings and compared with various traditional multi-objective optimization algorithms. The experimental results demonstrate that the proposed algorithm can achieve higher-quality Pareto fronts (PFs) and better convergence, indicating more equitable resource allocation and improved algorithmic effectiveness in addressing this issue. Moreover, these pre-prepared, high-quality solutions from PFs provide adaptability to varying requirements in signal collection scenarios.

## 1. Introduction

With the continuous maturation of Internet of Things (IoT) technology, the application of IoT sensors is becoming more prevalent [1,2]. A myriad of IoT devices are deployed globally, endowed with reconnaissance and monitoring functionalities, extensively applied in domains such as fire monitoring [3,4], ocean exploration [5,6], and wilderness search and rescue [7,8]. The smooth operation of these applications requires a reliable network coverage and sufficient channel rates. This is crucial because the data being transmitted often need to be conveyed to remote data processing centers [1]. However, the existing communication system-reliant base stations on the ground fall short of achieving global signal coverage. In regions such as the ocean, deserts, and areas afflicted by impaired or insufficient ground network facilities, data transmission becomes impeded and constrained.

To address the signal coverage challenges of IoT sensors in remote areas (IoRT), the integration of a Space–Air–Ground Network (SAGIN) has emerged as a predominant solution [9,10]. SAGIN involves the integration of diverse network resources, including ground networks, air networks, and satellite networks, into a unified system. This integration enables seamless connectivity and global-scale information transmission [11]. The overarching goal of this integrated network architecture is to enhance communication efficiency, stability, and coverage, thereby catering to the escalating demand for data transmission [12].

In existing research, air network nodes are commonly categorized into low-altitude platform (LAP) and high-altitude platform (HAP) [13]. LAP comprises airships, aircraft, and drones, while HAP encompasses balloons, solar-powered aircraft, and similar platforms. Notably, UAV-based LAPs offer advantages such as low energy consumption [14], high mobility [15], and extensive coverage [16]. Utilizing UAV-based relays can also establish line-of-sight (LoS) communication links for ground terminals. Space network nodes typically consist of low-Earth-orbit (LEO) satellites, medium-Earth-orbit (MEO) satellites, and geostationary-Earth-orbit (GEO) satellites. Among these, LEO satellites, characterized by the lowest altitude, offer advantages such as low latency and minimal propagation loss compared to MEO and GEO satellites.

Recent research works have focused significantly on improving signal coverage for terminal devices by integrating air and space communication layers and optimizing service quality. These research studies cover a range of aspects, including energy efficiency [17,18,19,20], bandwidth utilization efficiency [21,22], coverage range [23], communication security [24,25], and other parameters. Moreover, there is also a multitude of studies [26,27,28,29,30,31,32,33] dedicated to enhancing system channel capacity, focusing on optimizing resource allocation within SAGIN to subsequently optimize the channel capacity for overall systems or devices. For instance, Wang et al. [29] proposed a SAG-IoRT framework, where the authors jointly optimized UAV trajectory, smart device connection scheduling, and power control to enhance system capacity, addressing the challenges posed by the limited transmission power of remote devices. Furthermore, Ma et al. [26] presented an integrated UAV-LEO data collection framework for B5G Internet of Remote Things networks, focusing on optimizing IoT device bandwidth allocation, UAV 3D trajectory, and UAV power allocation to enhance data collection efficiency and minimize energy consumption. More descriptions in the literature are presented in the section about related works.

However, we observed some oversights in these studies. For instance, reference [29] optimized the overall system channel capacity, neglecting the varying demands and fairness among ground IoT devices, and it failed to rapidly adjust the resource allocation scheme in response to changes in ground demand. Regarding reference [26], a weighted function was optimized, which, due to the fixed weights between objectives, was not adaptable to scenarios where the requirements between objectives vary.

Therefore, this article simultaneously considers the aforementioned elements, aiming to delve into the challenges confronted by IoRT in terms of resource allocation fairness, demand-time variability, and diversity. This study encompasses a scenario involving multiple types of sensors, functioning as IoT devices, in data collection and uplink transmission. UAVs function as forwarding relays to transmit data collected by IoT devices to LEO satellites. By optimizing communication resources, including the deployment coordinates of drones, bandwidth allocation, and the association between drones and IoT devices, the minimum channel capacity of various IoT device types is maximized. Given limited communication resources, optimizing the channel capacity of different categories of IoT devices becomes a conflicting multi-objective optimization problem. Considering the involved variables, the problem also qualifies as a mixed-integer multi-objective optimization problem. In recent years, the MOEA/D algorithm has found widespread application in addressing diverse engineering problems with conflicting goals, spanning artificial intelligence [34,35], power engineering [36,37], the internet of vehicles [38], and more. To address this problem, this paper proposes an evolutionary algorithm for resource allocation based on the MOEA/D algorithm. This algorithm can generate a series of resource allocation solutions tailored to different scenarios of changing channel capacity demands for ground terminals. To the best of our knowledge, this type of scenario exploration has not been studied before.

The main contributions of this paper are summarized as follows:

(1) We conducted simulations on a SAGIN model, encompassing the ground IoT terminal-UAV layer and the UAV-LEO satellite layer. Diverse categories of IoT devices relay collected data to LEO satellites through drone relays. Within this model, we formulated a mixed-integer multi-objective optimization problem, wherein the deployment location of drones, bandwidth allocation, and connection selection between IoT devices and drones are collectively optimized. This optimization, in turn, maximizes the minimum channel capacity of various types of IoT devices.
(2) To address this non-convex problem, we propose a multi-objective evolutionary algorithm based on MOEA/D. The enhanced algorithm efficiently addresses the issue of equitable resource allocation among IoT devices of the same category within the SAGIN framework, while also exhibiting adaptability to the dynamic demand variations characteristic across various IoT device types.
(3) The robustness and superiority of the proposed algorithm are substantiated. We devised various scenarios of IoT device distribution, altering the number of IoT device types and UAV relays to validate the algorithm’s robustness. Additionally, the algorithm was compared with three other classic multi-objective optimization algorithms, affirming its superiority.

The remainder of the article is structured as follows: Some related works are presented in Section 2. In Section 3, we present the SAGIN model and articulate the multi-objective optimization problem through formulas. The algorithm proposed, elucidated in Section 4, attains fair and adaptive resource allocation for various IoT device types. Section 5 presents an overview of the proposed algorithm. Section 6 outlines the simulation experiments employed to validate the robustness and superiority of the proposed algorithm, accompanied by an analysis of the experimental results. Lastly, Section 7 provides a summary of the entire paper.

## 2. Related Works

Recent studies have placed considerable emphasis on enhancing the signal reach for end-user devices through the integration of terrestrial and space-based communication systems and the optimization of service quality. Cai et al. [17] presented a joint design of trajectory and resource allocation for energy-efficient secure UAV communication systems, optimizing the performance in the presence of multiple users and eavesdroppers through a non-convex optimization framework. The article [19] by Jia et al. investigated the optimization of UAV trajectory and data collection, assisted by LEO satellites, for energy-efficient operations in the context of 6G aerial access networks serving the IoRT. Shi et al. [21] proposed a joint optimization approach for gateway selection and resource allocation to enhance spectral efficiency in an SAG-IoRT network, addressing the challenges of cross-tier communication with UAVs as relays. Li et al. [23] investigated the enhancement of maritime coverage through the integration of UAVs with hybrid satellite–terrestrial networks, optimizing UAV trajectory and transmit power to maximize the minimum ergodic achievable rate under various constraints.

Some studies have concentrated on the allocation of communication resources, with a predominant focus on maximizing channel capacity optimization. For instance, Ma T et al. [26] focused on optimizing the trajectory and transmission power of UAVs, as well as selecting Earth-orbit satellites. They aimed to jointly optimize a weighted objective function, considering the total data uploaded by UAVs, energy consumption, and the overall average channel capacity of IoT devices. The optimization process involved the use of successive convex approximation and block coordinate descent techniques for effective problem resolution. Cao X et al. [27], with the goal of maximizing the total system throughput, determined the optimal probability of employing the SAGIN transmission scheme for ground terminals. In another study [28], a SAGIN framework based on a HAP as an air relay was proposed, with optimized resource allocation and HAP placement to maximize throughput for ground users. Addressing the maximization of channel capacity per unit timeslot, the work in [29] iteratively optimized connection scheduling, transmitting power, and UAV trajectory for ground intelligent terminals. Sun et al. [30] studied optimal three-dimensional trajectory design and resource allocation for UAVs to maximize the total system throughput within a given time period. Considering user fairness, reference [31] aimed to maximize the minimum throughput of users serviced by UAVs. In [32], a dual-objective optimization problem for a SAGIN scenario was proposed, considering both channel capacity consumption and maximization of ground terminal network coverage. A multi-objective evolutionary algorithm was adopted for its solution. Similarly, Xu Z et al. [39] employed airships for ground observation missions, utilizing a multi-objective evolutionary algorithm based on decomposition (MOEA/D) [40], dynamic weight vector (DW), and stable matching (STM) schemes to design airship deployment parameters. Jing Y et al. [41] built an Earth observation network platform using an airship and designed a multi-objective evolutionary algorithm to simultaneously optimize coverage and observation benefits. These related works are compiled in Table 1.

These studies focusing on optimizing channel capacity do not simultaneously account for the following factors:

(1) Equity in the allocation of communication resources among devices is not considered. Current methods optimize resource allocation based on the channel capacity of all IoT devices, potentially leading to substantial discrepancies in channel capacity among devices and inadequate capacities for certain IoT devices.
(2) Variations in the demand for channel capacity among different device types are overlooked. As different categories of IoT devices collect diverse data types, such as video data, audio data, infrared signal data, radar signal data, etc., distinct requirements for channel capacity exist among IoT device categories.
(3) The dynamic nature of channel capacity requirements for terminal equipment is not taken into account. The channel capacity needs of IoT devices are not static and may fluctuate with the rate of data collection. As demands change, new resource allocation schemes should be promptly adjusted.

Hence, this paper addresses the key elements collectively, exploring the IoRT’s resource allocation challenges, including fairness, dynamic demand, and diversity. We introduce various types of IoT devices to differentiate their distinct requirements, ensuring fairness among devices within the same category by optimizing for the minimum channel capacity. Additionally, we employ an enhanced multi-objective optimization algorithm to generate a range of feasible solutions that can rapidly adapt to changes in device channel capacity demands.

## 3. Syetem Model and Problem Formulation

### 3.1. System Model

**Figure 1:**
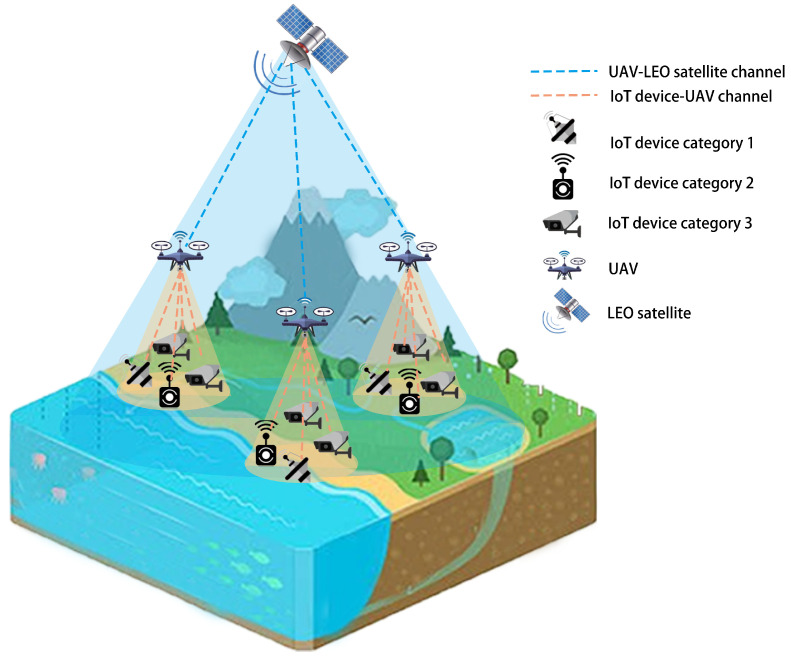
A three-layer network structure consisting of multiple IoT devices, UAVs, and an LEO satellite.

The system model addressed in this article is depicted as a three-layer network structure, comprising IoT devices located on the ground, UAV clusters deployed in the air, and LEO satellites. The corresponding schematic diagram is illustrated in Figure 1. Our emphasis is solely on the uplink transmission of the signal. Specifically, the data collected by ground IoT sensors are relayed by drones and transmitted to the LEO satellite in space. Subsequently, the LEO satellite transfers the data to the remote data center via the satellite network, a feature not considered in the model. *T* different types of IoT devices are taken into account, denoted as $I_{1},\ldots ,I_{t},\ldots ,I_{T}$, and their quantities are defined as $N_{1},\ldots ,N_{t},\ldots ,N_{T}$. The total number of IoT devices is defined as $\sum_{t=1}^{T}N_{t}=N^{I}$. We use $\left(x_{n}^{t},y_{n}^{t}\right)$ to denote the coordinate of the *n*-th device in the *t*-th category of IoT devices. The set of UAVs is represented by $U=[U_{1},\ldots ,U_{N^{U}}]$, where $N^{U}$ is the number of UAVs. The coordinate of the *n*-th drone is denoted as $L_{n}^{U}=\left(x_{n}^{U},y_{n}^{U},h^{U}\right)$, where $h^{U}$ is the fixed flight height of the drone. UAV relays are assumed to facilitate multiple-input multiple-output communications, enabling the simultaneous existence of multiple uplinks. The UAV relay is equipped with a robust signal receiver. Given the limited deployment range of IoT devices in this study, it is assumed that the UAV can effectively receive signals from all the ground IoT devices. The total available bandwidth of the entire communication system is defined as *B*. To prevent signal interference among IoT devices, we allocated the bandwidth into different shares, ensuring that the bandwidth resources occupied by each IoT device do not overlap. Correspondingly, we initially partition the bandwidth into *T* segments for various categories of IoT devices, with the ratio represented by $j=[j_{1},\ldots ,j_{t},\ldots ,j_{T}]$, $\sum_{t=1}^{T}j_{t}=1$. Next, we further subdivided the allocated bandwidth for IoT devices within each category. Specifically, for the *t*-th category of IoT devices, the bandwidth reallocation ratio is defined as $k^{t}=\left[k_{1}^{t},\ldots ,k_{n}^{t},\ldots ,k_{N_{t}}^{t}\right]$, $\sum_{t=1}^{N_{t}}k_{t}^{t}=1$. And the intra-class bandwidth allocation set for all categories of IoT devices is represented as $k=[k^{1},\ldots ,k^{T}]$. $a_{t}=\left[\alpha_{t1},\ldots ,\alpha_{tn},\ldots ,\alpha_{tN_{t}}\right]$ is utilized to denote the selection association of the *t*-th type of IoT devices to the drone, where $\alpha_{tn}\in \left\{1,\ldots ,N_{u}\right\}$, $n\in \{1,\ldots ,N_{t}\}$. In other words, the serial number of the associated drone for the *n*-th IoT device in $I_{t}$ is $\alpha_{tn}$. $A=[a_{1},\ldots ,a_{t},\ldots ,a_{T}]$ is used to represent the relationship between all types of IoT devices and UAVs. Simultaneously, $C_{tn}^{U}$ is defined as the number of IoT devices associated with the UAV selected by the *n*-th IoT device in $I_{t}$. It should be noted that one IoT device can only select one UAV for data transmission. Additionally, the transmit power of each ground IoT device is set to $P^{I}$. The total forwarding power of the drone is set to $P^{U}$, evenly distributed for signal forwarding to each connected IoT device. Furthermore, we consider an LEO satellite to stably receive the UAV’s forwarded signal, disregarding LEO satellite switching. The coordinate of the LEO satellite is denoted as $\left(x^{L},y^{L},h^{L}\right)$, where the altitude of LEO satellite is fixed at $h^{L}$.

### 3.2. Channel Model

The uplink transmission link of the system includes two parts: the uplink channel from the IoT device to the UAV, and the uplink channel from the UAV to the LEO satellite. Both channels are dominated by a line-of-sight (LoS) path, and the fading follows Rician fading [42]. For the *n*-th IoT device of the *t*-th category, we used $g_{n}^{I_{t}\to U_{\alpha_{tn}}}$ to denote the first-layer channel gain from the IoT device to the selected UAV, where $n\in \{1,\ldots ,N_{t}\}$. Additionally, $g_{n}^{U_{\alpha_{tn}}\to L}$ is employed to denote the gain of the second-layer channel in the uplink, specifically, the channel gain from the UAV to the LEO satellite. Drawing from refs. [29,42], considering that the carrier frequency significantly exceeds the system bandwidth, we assume that the calculation of channel gain is solely dependent on the communication distance. Therefore, the channel gain can be calculated by the following formulas:

(1) $$g_{n}^{I_{t}\to U_{\alpha_{tn}}}=\frac{g_{0}}{{\left(\sqrt{{\left(x_{n}^{t}-x_{\alpha_{tn}}^{U}\right)}^{2}+{\left(y_{n}^{t}-y_{\alpha_{tn}}^{U}\right)}^{2}+h^{U^{2}}}\right)}^{2}}$$

(2) $$g_{n}^{U_{\alpha_{tn}}\to L}=\frac{g_{0}}{{\left(\sqrt{{\left(x_{\alpha_{tn}}^{U}-x^{L}\right)}^{2}+{\left(y_{\alpha_{tn}}^{U}-y^{L}\right)}^{2}+h^{L^{2}}}\right)}^{2}}$$

where $g_{0}$ is the channel gain per unit distance. $\gamma_{n}^{I_{t}\to U_{\alpha_{tn}}}$ is expressed as the channel signal-to-noise ratio from the *n*-th device in $I_{t}$ to the associated UAV, and $\gamma_{n}^{U_{\alpha_{tn}}\to L}$ is expressed as the channel signal-to-noise ratio from the selected UAV to the LEO satellite. Furthermore, we assume that the power spectral density of additive white Gaussian noise (AWGN) is $N_{0}$. $B_{n}^{t}=Bj_{t}k_{n}^{t}$ is used to represent the bandwidth occupied by the *n*-th device in $I_{t}$. For the *n*-th UAV, as its forwarding power is uniformly distributed among each connected IoT device, the forwarding power allocated to the *n*-th device is $P_{n}^{t}=P^{U}/C_{tn}^{U}$. Therefore, $\gamma_{n}^{I_{t}\to U_{\alpha_{tn}}}$ and $\gamma_{n}^{U_{\alpha_{tn}}\to L}$ can be obtained by the following formulas:

(3) $$\gamma_{n}^{I_{t}\to U_{\alpha_{tn}}}=\frac{P^{I}g_{n}^{I_{t}\to U_{\alpha_{tn}}}}{Bj_{t}k_{n}^{t}N_{0}}$$

(4) $$\gamma_{n}^{U_{\alpha_{tn}}\to L}=\frac{P^{U}g_{n}^{U_{\alpha_{tn}}\to L}}{Bj_{t}k_{n}^{t}C_{tn}^{U}N_{0}}$$

According to the principles of amplification, forwarding relay, and Shannon’s formula, the uplink channel transmission rate of the *n*-th device in $I_{t}$ can be calculated using the following formula:

(5) $$R_{n}^{t}=B_{n}^{t}log_{2}\left(1+\frac{\gamma_{n}^{I_{t}\to U_{\alpha_{tn}}}\gamma_{n}^{U_{\alpha_{tn}}\to L}}{1+\gamma_{n}^{I_{t}\to U_{\alpha_{tn}}}+\gamma_{n}^{U_{\alpha_{tn}}\to L}}\right)$$

For easy reference, a notation summary of the system model is presented in Table 2.

### 3.3. Problem Formulation

In this section, a multi-objective optimization problem is introduced to address the uplink channel resource allocation challenge. The decision variables for this multi-objective optimization problem encompass UAV deployment locations, bandwidth allocation ratios for different categories of IoT devices, bandwidth allocation ratios for IoT devices within a category, and UAV-IoT device selection association. The objective is to maximize the minimum channel capacity within each category of IoT devices, with the the number of objective functions equal to the number of IoT device categories. The multi-objective optimization problem can be formulated as follows:

(6) $$\max_{L}\left\{\begin{array}{cc} min(R_{n}^{1}), & \forall n\in \{1,\ldots ,N_{1}\}\\ \ldots \\ min(R_{n}^{t}), & \forall n\in \left\{1,\ldots ,N_{t}\right\},\forall t\in \left\{1,\ldots ,T\right\}\\ \ldots \\ min(R_{n}^{T}), & \forall n\in \{1,\ldots ,N_{T}\} \end{array}\right.$$

subject to

(7) $$L=[L_{n}^{U},j,k,A]$$

(8) $$\begin{array}{cc} \; & \sum_{t=1}^{T}j_{t}=1,\forall t\in \left\{1,\ldots ,T\right\} \end{array}$$

(9) $$\begin{array}{cc} \; & \sum_{t=1}^{T}k_{t}=1,\forall t\in \left\{1,\ldots ,T\right\} \end{array}$$

(10) $$\begin{array}{cc} \; & \sum_{n=1}^{N_{t}}\sum_{t=1}^{T}P_{n}^{t}=P^{U},\forall n\in \left\{1,\ldots ,N_{t}\right\},\forall t\in \left\{1,\ldots ,T\right\} \end{array}$$

(11) $$\begin{array}{cc} \; & j_{t},k_{t}\in \left[0,1\right],\forall n\in \left\{1,\ldots ,N_{t}\right\},\forall t\in \left\{1,\ldots ,T\right\} \end{array}$$

In the formula, min() denotes selecting the minimum value. $L=[L_{n}^{U},j,k,A]$ represents all the decision variables of this optimization problem. Formulas (8) and (9) indicate that all bandwidth resources will be fully utilized to prevent bandwidth waste. Formula (10) states that the forwarding power of the UAV is fully allocated, with the total power limited to $P^{U}$. Formula (11) specifies that the distribution ratio value is constrained to be between 0 and 1. The decision variable L includes a mix of continuous and discrete elements. Given the nonlinearity of the objective function, this constitutes a mixed-integer nonlinear programming (MINLP) problem, which is non-convex [24,43]. In the presence of limited resources, the optimization among goals is contradictory and conflicting. To accommodate changes in the needs of IoT devices and facilitate rapid resource allocation adjustments, a set of feasible solutions should be pre-established. Coincidentally, multi-objective evolutionary algorithms excel at solving this type of problem. Therefore, in the next section, the solution using the improved MOEA/D algorithm is introduced.

## 4. Algorithm Design

### 4.1. Problem Analysis

**Figure 2:**
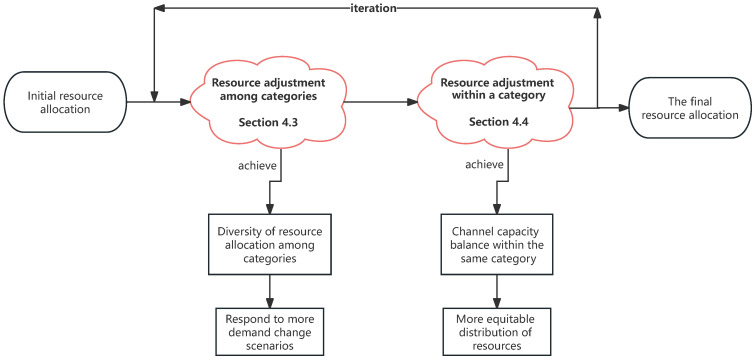
Resource allocation logic diagram.

This resource allocation problem is first logically analyzed. The resource allocation problem involves two steps: one is the resource allocation of different categories of IoT devices, and the other is the resource allocation of IoT devices within the same category. The desired outcomes for the final result are twofold. First, the final Pareto solution set should encompass as many solutions as possible to accommodate a variety of demand scenarios. Second, the channel capacity among the same type of IoT devices should be as close as possible to achieve fair resource allocation and minimize waste. Therefore, the entire resource allocation logic diagram can be summarized in Figure 2. In general, the purpose of resource adjustment among categories is to diversify the proportion of resources occupied by different categories, enabling diversified minimum channel capacity values after a fair allocation of resources, thereby enriching the Pareto solution set. The purpose of resource adjustment within a category is to bring the channel capacity among the same category of IoT devices as close as possible, achieving fair resource allocation.

**Figure 3:**
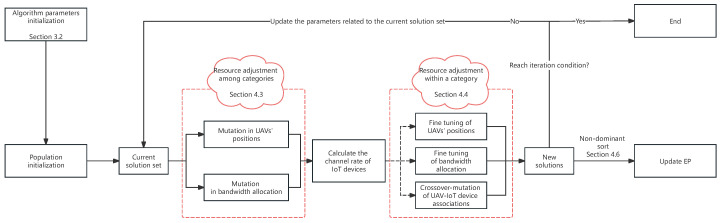
New solution generation logic of MOEA/D-MFAR.

To address this challenge, this paper proposes an improved multi-objective evolutionary algorithm based on decomposition for a multi-party fair allocation of resources (MOEA/D-MFAR). In all evolutionary algorithms, the crux lies in the generation mechanism of new solutions. Combining the resource allocation logic discussed earlier and the types of resources involved in this paper, the flow of MOEA/D-MFAR including new solution generation logic is summarized in Figure 3. Initially, for resource adjustment among categories, only continuous variables are probabilistically adjusted. Specifically, the deployment positions of UAVs and the allocation ratio of bandwidth are adjusted. This is because adjusting discrete variables might lead to abrupt changes in the target value, which is detrimental to the convergence of the optimization process. Subsequently, we calculated and identified devices with the maximum and minimum channel capacity within each type of IoT device under the current resource allocation. Finally, for each type of IoT device, resources are probabilistically transferred from the device with the largest channel capacity to the device with the smallest channel capacity. Concerning the discrete variable UAV-IoT device correlation, we employed the crossover–mutation operation for probabilistic adjustments, subsequently influencing the allocation of resources between classes.

### 4.2. Parameter Initialization in MOEA/D-MFAR

The algorithm parameters are initialized following the fundamental MOEA/D framework. Firstly, *M* is defined as the population size. Secondly, *M* uniformly distributed unit vectors $\lambda^{1},\ldots ,\lambda^{M}$ are generated using Chebyshev distance. Thirdly, for each unit vector $\lambda^{m}$, *M* neighborhoods $B\left(m\right)=i_{1},\ldots ,i_{s}$ are determined based on the Chebyshev distance. *S* is the size of the neighbor, and $i_{1},\ldots ,i_{s}$ is the subscript of the *T* unit vectors closest to $\lambda^{m}$. Other symbols are defined as follows:

(1) *Q* is defined as the number of objective functions.
(2) $z=(z_{1},\ldots ,z_{q},\ldots ,z_{Q})$, where $z_{q}$ is the best performance value of the *q*-th objective function.
(3) $FV_{1},\ldots ,FV_{m},\ldots ,FV_{M}$, where $FV_{m}=\left[fv_{1}\left(\Gamma_{m}\right),\ldots ,fv_{q}\left(\Gamma_{m}\right),\ldots ,fv_{Q}\left(\Gamma_{m}\right)\right]$, and $fv_{q}\left(\Gamma_{m}\right)$ is the value of the *m*-th individual $\Gamma_{m}$ on the *q*-th objective function.
(4) The target value calculation formula of the *m*-th sub-problem is as follows:
  (12) $$g^{te}\left(\Gamma_{m}|\lambda^{m},z\right)=\max_{1\leq q\leq Q}\left\{\lambda_{q}^{m}|fv_{q}\left(\Gamma_{m}\right)-z_{q}\right\}$$
(5) EP is defined as the set of feasible solutions eventually obtained by the algorithm, and PF is the set of target values corresponding to the solutions in EP.

### 4.3. Resource Adjustment Among Categories

As mentioned earlier, resource adjustment among categories contributes to obtaining more feasible solutions, comprising two aspects: the mutation in UAVs’ positions and the mutation in bandwidth allocation.

#### 4.3.1. Mutation in UAVs’ Positions

The mutation probability of each UAV is set to $PB_{um}$. For the *n*-th UAV, $\forall n\in \{1,\ldots ,N^{U}\}$, the formulas for the mutation operation are described as follows:

(13) $$\begin{array}{cc} x_{n}^{u^{\prime }} & =x_{n}^{u}+e_{n}^{x} \end{array}$$

(14) $$\begin{array}{cc} y_{n}^{u^{\prime }} & =y_{n}^{u}+e_{n}^{y} \end{array}$$

(15) $$\begin{array}{cc} e_{n}^{x} & =random(-c_{1}x_{max},c_{1}x_{max}) \end{array}$$

(16) $$\begin{array}{cc} e_{n}^{y} & =random(-c_{1}y_{max},c_{1}y_{max}) \end{array}$$

where $e_{n}^{x}$ and $e_{n}^{y}$ are the horizontal and vertical coordinates of the *n*-th UAV. random(a,b) means that the value will be randomly determined between *a* and *b*. $x_{n}^{u}$ and $y_{n}^{u}$ represent the coordinates before mutation, while $x_{n}^{u^{\prime }}$ and $y_{n}^{u^{\prime }}$ represent the coordinates after mutation. $x_{max}$ and $y_{max}$, respectively, denote the maximum horizontal and vertical distances of the IoT device deployment area. Parameter $c_{1}$ ranges between 0 and 1 and is set to 0.1. Additionally, if the adjusted coordinates exceed the limits of $x_{max}$ and $y_{max}$, the coordinates will be adjusted to the edge values of the area.

#### 4.3.2. Mutation in Bandwidth Allocation

**Figure 4:**
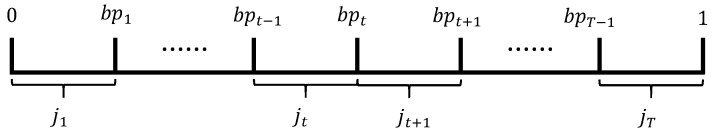
Bandwidth allocation model.

As the bandwidth is fully allocated, the bandwidth allocation model is depicted in Figure 4, and the allocation process can be perceived as the determination of breakpoints on line segments. For the scenario including *T* types of IoT devices, there exist *T*-1 breakpoints. The mutation of bandwidth allocation can be regarded as the movement of breakpoints. Each breakpoint movement is assigned a probability of execution, denoted as $PB_{bdm}$. Specifically, for the *t*-th breakpoint, t∈{1,…,T}, the mutation operation is descried as follows:

If ρ<0.5, the *t*-th breakpoint moves left:

(17) $$\begin{array}{cc} j_{t}^{\prime } & =j_{t}-e_{bd}^{t} \end{array}$$

(18) $$\begin{array}{cc} j_{t+1}^{\prime } & =j_{t+1}+e_{bd}^{t} \end{array}$$

(19) $$\begin{array}{cc} e_{bd}^{t} & =random(0,c_{2}\times j_{t}) \end{array}$$

Otherwise, the *t*-th breakpoint moves right:

(20) $$\begin{array}{cc} j_{t+1}^{\prime } & =j_{t+1}-e_{bd}^{t} \end{array}$$

(21) $$\begin{array}{cc} j_{t}^{\prime } & =j_{t}+e_{bd}^{t} \end{array}$$

(22) $$\begin{array}{cc} e_{bd}^{t} & =random(0,c_{2}\times j_{t+1}) \end{array}$$

where $K_{t}^{\prime }$ is the new bandwidth allocation ratio for the *t*-th category of IoT devices. ρ∼U(0,1) is used to determine the moving direction of the breakpoint. $e_{bd}^{t}$ is the movement amount for the bandwidth breakpoint. When the breakpoint moves left, it is randomly determined within $\left(0,c_{2}\times K_{t}\right)$. When it moves right, it is randomly determined within $\left(0,c_{2}\times K_{t+1}\right)$. Parameter $c_{2}$ is defined between 0 and 1; here, it is taken as 0.5. It should be noted that the breakpoints need to be moved sequentially starting from $bp_{1}$. In Formulas (17) and (19), $j_{t}$ is the bandwidth allocation ratio of the new *t*-th-category IoT determined after the movement of $bp_{t-1}$.

### 4.4. Resource Adjustment Within a Category

Resource adjustment within a category aims to achieve channel capacity balance. It comprises three components: fine-tuning of UAVs’ positions, fine-tuning of bandwidth allocation, and crossover–mutation of UAV-IoT device association.

#### 4.4.1. Fine-Tuning of UAVs’ Positions

The closer the drone is to the IoT device, the smaller the channel loss between the two, resulting in higher channel capacity. Therefore, the purpose of fine-tuning the UAV positions is to bring them closer to the IoT devices with the minimum channel capacity. For the *n*-th UAV, the *t*-th-category IoT device,$\forall n\in \{1,\ldots ,N^{U}\}$, ∀t∈{1,…,T}, this fine-tuning can be expressed by the following formulas:

(23) $$\begin{array}{cc} x_{n}^{u^{\prime \prime }} & =x_{n}^{u^{\prime }}+d_{nt}^{x}f_{bt}^{x} \end{array}$$

(24) $$\begin{array}{cc} y_{n}^{u^{\prime \prime }} & =y_{n}^{u^{\prime }}+d_{nt}^{y}f_{bt}^{y} \end{array}$$

and

(25) $$d_{nt}^{x}=\left(x^{I_{tmin}}-x_{n}^{u^{\prime }}\right)/\left|x^{I_{tmin}}-x_{n}^{u^{\prime }}\right|$$

(26) $$\begin{array}{cc} d_{nt}^{y} & =\left(y^{I_{tmin}}-y_{n}^{u^{\prime }}\right)/\left|y^{I_{tmin}}-y_{n}^{u^{\prime }}\right| \end{array}$$

(27) $$\begin{array}{cc} f_{nt}^{x} & =\mathrm{random}(0,c_{3}\times r\times |x^{I_{tmin}}-x_{n}^{u^{\prime }}|) \end{array}$$

(28) $$\begin{array}{cc} f_{nt}^{y} & =\mathrm{random}(0,c_{3}\times r\times |y^{I_{tmin}}-y_{n}^{u^{\prime }}|) \end{array}$$

where $x^{I_{tmin}}$ and $y^{I_{tmin}}$ represent the horizontal and vertical coordinates of the device with the minimum channel capacity in $I_{t}$, and $x_{n}^{u^{\prime }}$ and $y_{n}^{u^{\prime }}$ represent the horizontal and vertical coordinates of the UAV after fine-tuning. $c_{3}$ is a value between [0, 1], with a default value of 0.5. *r* is also a dynamic parameter between 0 and 1, which is the decay rate parameter. The determination of the value can be understood in the subsequent introduction of the decay rate mechanism, presented in Section 4.5. Through continuous iteration, the position of the UAV can persistently approach the IoT device with the minimum channel capacity.

#### 4.4.2. Fine-Tuning of Bandwidth Allocation

The more bandwidth an IoT device occupies, the greater its channel rate. Therefore, in the process of fine-tuning, bandwidth is reallocated from IoT devices with the highest channel capacity to IoT devices with the lowest channel capacity. For $N_{t}$ IoT devices, and t∈{1,…,T}, the bandwidth fine-tuning can be described as follows:

(29) $$\begin{array}{cc} k_{t}^{Rmin^{\prime }} & =k_{t}^{Rmin}+f_{t}^{bd} \end{array}$$

(30) $$\begin{array}{cc} k_{t}^{Rmax^{\prime }} & =k_{t}^{Rmax}-f_{t}^{bd} \end{array}$$

(31) $$\begin{array}{cc} f_{t}^{bd} & =\mathrm{random}(0,c_{4}\times r\times k_{t}^{cmax}) \end{array}$$

where $k_{t}^{Rmin}$ and $k_{t}^{Rmax}$ represent the proportion of bandwidth occupied by the IoT devices currently having the minimum and maximum channel rates in $I_{t}$. $f_{t}^{bd}$ indicates that the fine-tuning amount of bandwidth is also the transfer amount, which is randomly determined within $\left(0,c_{4}\times r\times k_{t}^{Rmax}\right)$. $c_{4}$ is a parameter between 0 and 1 and is defined as 0.5 here. Similarly, *r* as a decay rate parameter will be introduced later.

#### 4.4.3. Crossover–Mutation of UAV-IoT Device Association

**Figure 5:**
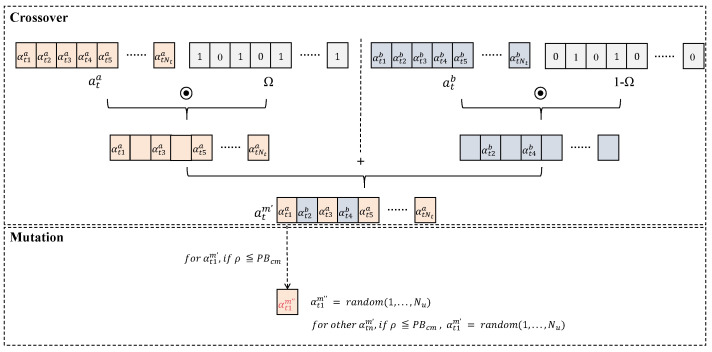
Crossover–mutation operation example diagram.

For the UAV-IoT device association of the *t*-th-category IoT device, due to the absence of a clear direction for the adjustment of resources from high to low, a crossover–mutation method is utilized for optimization. Crossover mimics biological reproduction in genetic algorithms, generating offspring by blending the genetic material of multiple parents. Mutation introduces randomness into an individual’s genome to diversify the population and avoid early convergence to local optima. The specific implementation methods are illustrated below, and the case of crossover and mutation is also illustrated in Figure 5.

##### Crossover

**Figure 6:**
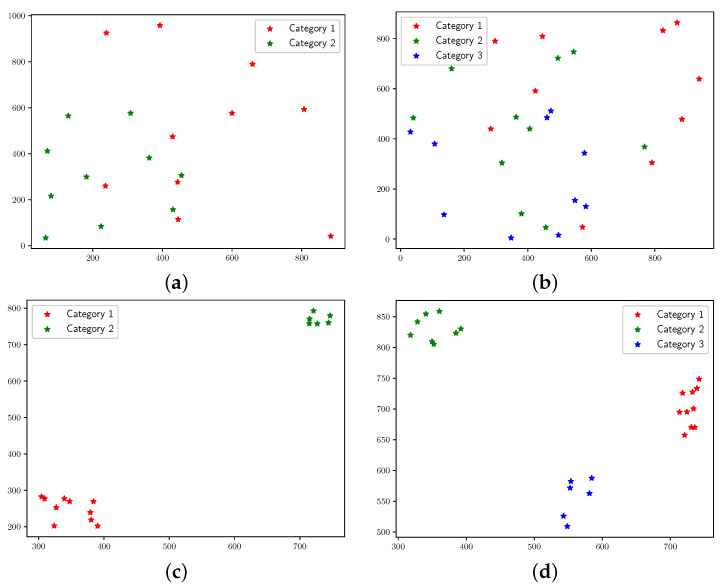
Distribution of IoT devices in different scenarios. (**a**) Distribution of IoT devices in scenarios 1 and 2. (**b**) Distribution of IoT devices in scenarios 5 and 6. (**c**) Distribution of IoT devices in scenarios 3 and 4. (**d**) Distribution of IoT devices in scenarios 7 and 8.

For the *m*-th individual, two indexes, *a* and *b*, are randomly selected from the neighborhood B(m). $a_{t}^{a}=\left[\alpha_{t1}^{a},\ldots ,\alpha_{tn}^{a},\ldots ,\alpha_{tN_{t}}^{a}\right]$ and $a_{t}^{b}=\left[\alpha_{t1}^{b},\ldots ,\alpha_{tn}^{b},\ldots ,\alpha_{tN_{t}}^{b}\right]$ are used to represent the UAV selection situation of the *t*-th-category IoT device in the *a*-th and *b*-th individuals. Therefore, the crossover operation can be expressed by the following formula, where Ω is a randomly generated vector containing only 0 and 1, and representing element-based multiplication. The symbol ⊙ represents the dot product operation, illustrated in Figure 6. After the crossover operation, the new UAV-IoT device association $a_{t}^{m^{\prime }}=\left[\alpha_{t1}^{m^{\prime }},\ldots ,\alpha_{tn}^{m^{\prime }},\ldots ,\alpha_{tN_{t}}^{m^{\prime }}\right]$ for $I_{t}$ is obtained.

(32) $$a_{t}^{m^{\prime }}=a_{t}^{a}⊙\Omega +a_{t}^{b}⊙\left(1-\Omega \right)$$

##### Mutation

The mutation operation occurs after the crossover operation. For the *n*-th IoT device in $I_{t}$, $n\in \{1,\ldots ,N_{t}\}$, we performed the mutation operation with the mutation probability $PB_{cm}$. Finally, $C_{t}^{m^{\prime \prime }}=\left[\alpha_{t1}^{m^{\prime \prime }},\ldots ,\alpha_{tn}^{m^{\prime \prime }},\ldots ,\alpha_{tN_{t}}^{m^{\prime \prime }}\right]$ is the result after crossover–mutation operation, representing UAV-IoT device association for $I_{t}$.

(33) $$\begin{array}{cc} \alpha_{tn}^{m^{\prime \prime }} & =\mathrm{random}\left(1,\ldots ,N_{u}\right),\mathrm{if}\;\rho \leq PB_{cm} \end{array}$$

(34) $$\begin{array}{cc} \alpha_{tn}^{m^{\prime \prime }} & =\alpha_{tn}^{m^{\prime }},\mathrm{otherwise} \end{array}$$

### 4.5. Decay Rate Mechanism

The degree of fine-tuning needs to decrease as the iteration progresses. This reduction aids in accelerating convergence during the early stages of iteration and prevents oscillations near the optimal solution in the later stages. Therefore, a decay rate mechanism is employed, involving the introduction of the dynamic parameter *r*. The parameter *r* is determined using the following formula:

(35) $$r=b^{epoch}\times r_{i}$$

where $r_{i}$ is the initial decay rate, set to 1. *b* is the base between 0 and 1, and epoch is the current iteration number.

### 4.6. Evaluating Solution Dominance

Once a new solution is introduced into EP, it is necessary to filter the feasible solutions within EP to eliminate those that are dominated, ensuring that all solutions in EP are mutually non-dominated. Specifically, a solution $\Gamma_{i}$ is dominated by another solution $\Gamma_{j}$ if it simultaneously satisfies the following two conditions:

(1) Non-inferiority: The solution $\Gamma_{j}$ is not worse than the solution $\Gamma_{i}$ in all objectives, that is, $fv_{q}\left(\Gamma_{j}\right)\leq fv_{q}\left(\Gamma_{i}\right),\forall q\in \left\{1,\ldots ,Q\right\}$.
(2) Dominance: The solution $\Gamma_{j}$ is strictly better than the solution $\Gamma_{i}$ in at least one objective, that is, $fv_{q}\left(\Gamma_{j}\right)<fv_{q}\left(\Gamma_{i}\right),\exists q\in \left\{1,\ldots ,Q\right\}$.

## 5. Overview of Algorithm

The combination of the aforementioned Section 4.3, Section 4.4 and Section 4.5 is considered as a novel solution generation mechanism for MOEA/D-MFAR. Corresponding to Figure 3, the complete algorithm flow is delineated in Algorithm 1.

The complexity of Algorithm 1 is mainly derived from Step 3. The complexity of 3.1 is O(M), and the complexity of 3.1.1 is related to the number of UAVs $N^{U}$ as well as the types of IoT devices *T*, which is $O\left(MN^{U}\right)+O\left(MT\right)$. The complexity of 3.1.2 is $O(MN^{I})$. The complexity of 3.1.3 includes the fine-tuning of UAV positions, the fine-tuning of bandwidth, as well as the crossover and mutation operations of UAV-IoT association, with the complexities of these four parts being $O\left(MN^{U}\right)+O\left(MT\right)+O\left(MT\right)+O\left(MN^{I}\right)$, respectively. For 3.2, the complexity is related to the number of objectives *Q*, which is O(QM). For 3.3, the complexity is related to the field size *S* and the number of objectives *Q*, which is O(QSM). For 3.4, assuming that each individual can generate new and better solutions, the worst-case complexity is $O(M^{2})$. Considering that $N^{I}$ is larger than *T* and $N^{U}$, the worst-case complexity for Step 3 is $O\left(MNI\right)+O\left(QMS\right)+O\left(M^{2}\right)$. Taking into account the total number of iterations *E*, the worst-case complexity of the algorithm is $O(EMN^{I}+EMQS+EQM^{2})$. Under normal circumstances, $N^{I}$, *Q*, and *S* are all smaller than *M*, so the main complexity of the algorithm comes from the non-dominated sorting, which is $O(EQM^{2})$. This is also the current state of most multi-objective optimization algorithms based on non-domination.
**Algorithm** **1** The flow of MOEA/D-MFAR algorithm. 1:(1) **INPUT** : 2:    *E*: Number of iteration steps, *M*: population size; 3:    *S*: The size of the neighborhoods; some scene parameters; 4:(2) **INITIALIZATION** : 5:    (2.1) Initialize *EP* = ∅, and *PF* = ∅; 6:    (2.2) Randomly initialize *M* individuals; 7:    (2.3) Calculate $FV_{1},\ldots ,FV_{M}$; 8:    (2.4) Initialize $z^{*}=\left(z_{1},\ldots ,z_{Q}\right)$; 9:    (2.5) Generate $\lambda^{1},\ldots ,\lambda^{M}$, and B(m);10:(3) **UPDATE** :11:**for** m=1,…,M **do**12:    (3.1) Generate new solutions $\Gamma_{m}^{\prime }$ according to 4.3 and 4.4;13:    (3.2) Update $z^{*}$: For each q=1,…,Q, if $fv_{q}\left(\Gamma_{m}^{\prime }\right)>z_{q},z_{q}=fv_{q}\left(\Gamma_{m}^{\prime }\right)$;14:    (3.3) Update field solutions in B(m): For each sequence number i∈B(m), if $g^{te}\left(\Gamma_{m}^{\prime }|\lambda^{i},z^{*}\right)\leq g^{te}\left(\Gamma_{m}|\lambda^{i},z^{*}\right)$, then set $\Gamma_{m}=\Gamma_{m}^{\prime }$ and $FV_{m}=FV_{m}^{\prime }$;15:    (3.4) Update *EP* and *PF*:16:    $EP\leftarrow EP\cup \{\Gamma_{m}^{\prime }\}$                       ▹ Add $\Gamma_{m}^{\prime }$ to *EP*17:    Remove dominated solutions from *EP*18:    PF←UpdatePF(EP)               ▹ Update *PF* based on the new *EP*19:**end for**20:**UNTIL** the termination condition is met.21:**OUTPUT** *EP* the *PF*.

## 6. Simulation Results

### 6.1. Experimental Settings

The robustness and effectiveness of the proposed algorithm will be validated in this section. In the context of real-world applications, the deployment of UAVs and IoT devices is subject to variability in terms of quantity, device types, and geographical distribution, contingent upon the specific mission requirements. To enhance the realism of the simulation and to assess the robustness of the proposed algorithm, it is imperative to consider a diverse range of scenarios. Hence, multiple scenarios are introduced and listed in Table 3, totaling eight scenarios. Two distinct IoT-device-distribution characteristics are presented in Figure 6. Specifically, scenarios 1, 2, 5, and 6 exhibit sparse IoT device distribution, while scenarios 3, 4, 7, and 8 feature dense distribution. The number of IoT device types is set to 2 and 3, addressing dual-objective and three-objective optimization, respectively. For three-objective problems (scenarios 5–8), the expansion of solution space dimensions results in a wider spread of optimal solutions, elevating search difficulty. Thus, the population size *M* is increased to enhance solution space exploration. The number of different types of IoT devices varies in each scenario, as detailed in Table 3. For the number of UAVs, 1 and 3 are set. It is important to note that when the number of UAV is 1, there is no need to consider the association selection of UAV relay by IoT devices. In scenarios with three UAVs (scenarios 2, 4, 6, and 8), the search for optimal solutions becomes more challenging. To address this, the base of decay rate *b* is decreased to improve search precision in the solution space, with its value determined through rigorous pre-experimental analysis. Other simulation parameters are outlined in Table 4. The environmental parameters were set based on references from the relevant literature [29,44,45], while the algorithm parameters were determined through multiple preliminary experiments to obtain relatively reasonable values. All simulation experiments were conducted utilizing Python 3.9, with the subsequent visualization of results executed through the Matplotlib library. Furthermore, portions of the experimental content in this paper have previously appeared in a conference paper [46]. However, the scope was limited to just two experimental scenarios, two comparative algorithms, two objective functions, and a single number of UAVs, neglecting the correlation between UAVs and IoT devices.

In order to verify the effectiveness of the algorithm, MOEA/D-MFAR was compared with three classic multi-objective optimization algorithms. The benchmark algorithms used in our study are as follows:

- MOEA/D: As originally proposed [40], it employs a genetic algorithm for solution generation. This algorithm is also applied to similar SAGIN [32] scenarios.
- NSGA-II [47]: The Non-dominated Sorting Genetic Algorithm II (NSGA-II), which includes an elite screening mechanism and a rapid non-dominated sorting technique, is utilized to ensure convergence while preserving search efficiency. This algorithm is also integrated into comparative experiments within the context of airship observation ground missions [39].
- MOPSO [48]: Multi-objective Particle Swarm Optimization (MOPSO), a variant of the Particle Swarm Optimization algorithm, is specifically designed to tackle multi-objective problems. In a previous application [49], it was utilized to allocate communication resources for UAV relays to ships.

### 6.2. Verification of Decay Rate Mechanism

**Figure 7:**
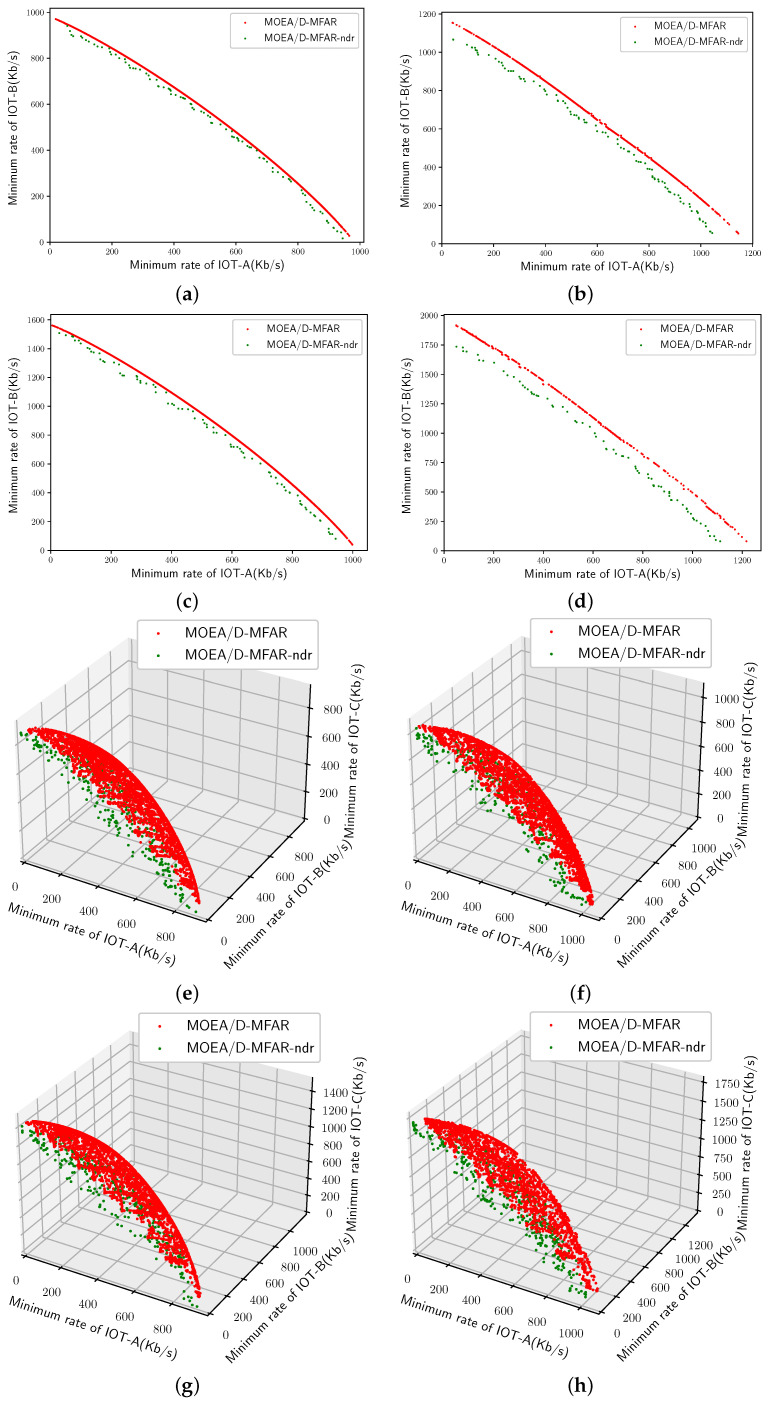
PFs obtained by MOEA/D-MFAR and MOEA/D-MFAR-ndr. (**a**) Scenario 1. (**b**) Scenario 2. (**c**) Scenario 3. (**d**) Scenario 4. (**e**) Scenario 5. (**f**) Scenario 6. (**g**) Scenario 7. (**h**) Scenario 8.

Initially, we assessed the impact of the decay rate mechanism on the overall evolutionary process. Eight scenarios were examined through two sets of experiments: MOEA/D-MFAR with the decay rate mechanism and MOEA/D-MFAR without the decay rate mechanism (MOEA/D-MFAR-ndr). The parameter configurations for these experiments align with the specifications outlined in Table 4. The Pareto fronts (PFs) resulting from the experiments across the eight simulation scenarios are depicted in Figure 7. The red scattered points denote the performance of MOEA/D-MFAR, while the green scattered points represent MOEA/D-MFAR-ndr performance. Evidently, no matter what scenario settings, the presence of the decay rate mechanism enhances the performance of the obtained PFs. The PFs comprising red scatter points consistently appear closer to the top-right corner compared to their green counterparts. Notably, the red PFs entirely dominate the green PFs, signifying that the IoT devices achieve a higher channel rate with the aid of the decay rate mechanism. Furthermore, the red frontiers display a noticeably denser distribution than the green ones, indicating a larger number of feasible solutions. These solutions accommodate varying demand scenarios. To quantify the performance of the obtained PFs, we computed and presented the hypervolume (HV) values and the number of solutions in Table 5. HV serves as a quantitative index assessing the quality of PFs, with larger HV values indicating superior performance. All HV calculations herein use (0, 0) as the reference point. The comparative analysis reveals consistently smaller HV for MOEA/D-MFAR-ndr than for MOEA/D-MFAR, emphasizing the advantageous impact of the decay rate mechanism, and the number of feasible solutions will be greatly increased, aided by the decay rate mechanism.

**Figure 8:**
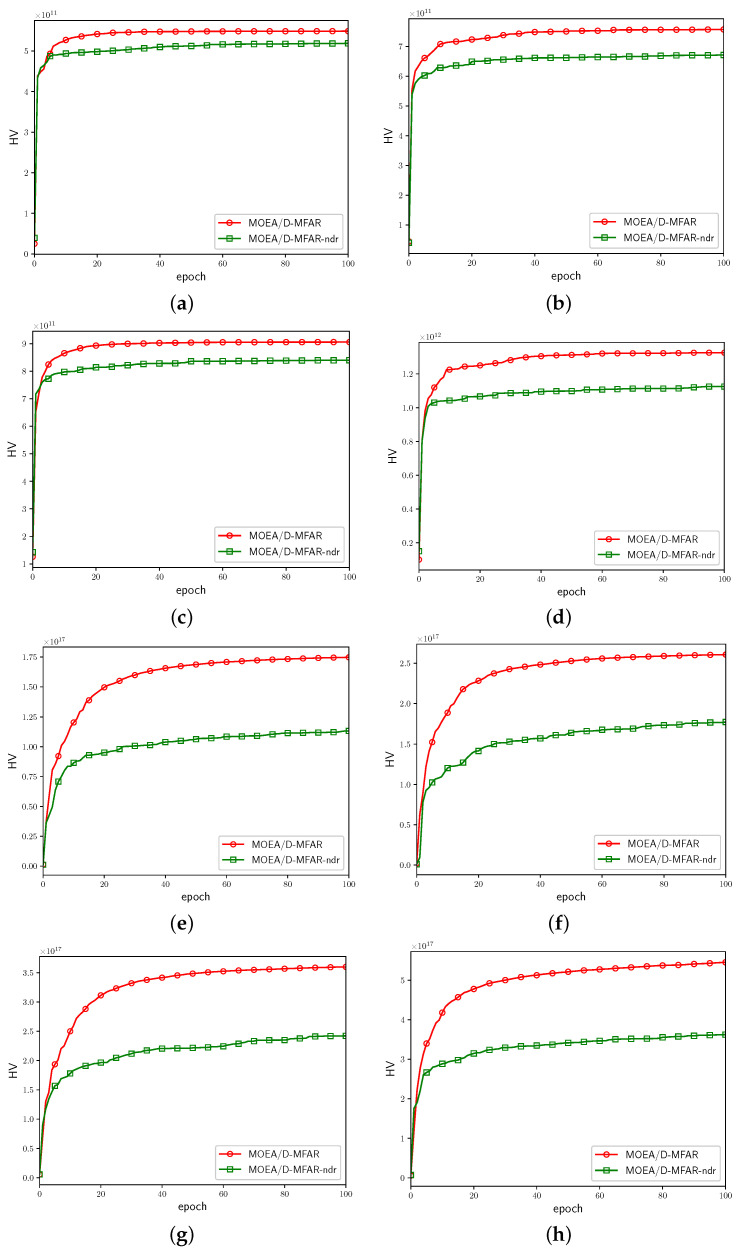
HV curves of MOEA/D-MFAR and MOEA/D-MFAR-ndr in eight scenarios. (**a**) Scenario 1. (**b**) Scenario 2. (**c**) Scenario 3. (**d**) Scenario 4. (**e**) Scenario 5. (**f**) Scenario 6. (**g**) Scenario 7. (**h**) Scenario 8.

The HV values corresponding to each epoch are logged and illustrated in Figure 8. This approach allows for the visualization of PF performance and facilitates the observation of the convergence process. The red curves depict the HV fluctuations for the algorithm incorporating a decay rate mechanism, whereas the green curves represent the algorithm devoid of a decay rate mechanism. Evidently, in comparison to the red curves, the red curves ultimately attain a higher HV value, indicating a faster convergence speed.

Moreover, to assess the fairness of resource allocation among IoT devices of the same type, we computed the average standard deviation of the channel rate (ASDR) and present the results in Table 6. For the *t*-th type of IoT devices, $n\in \{1,\ldots ,N_{t}\}$, the ASDR calculation formula is described as Formula (36), where $N_{s}$ is the number of solutions in EP, and $n_{s}$ is the sequence number of the solution. A smaller ASDR indicates closer channel rates among devices, reflecting a fairer resource allocation. Notably, the incorporation of decay rate mechanisms contributes to a reduction in channel rate differences among similar IoT devices.

(36) $$ADSR=\frac{1}{N_{s}}\sum_{n_{s}=1}^{N_{s}}\left(\frac{1}{T}\sum_{t=1}^{T}\left(\sqrt{\frac{1}{N_{t}}\sum_{n=1}^{N_{t}}\left(R_{n}^{t}-\frac{1}{N_{t}}\sum_{n=1}^{N_{t}}R_{n}^{t}\right)}\right)\right)$$

### 6.3. Algorithm Comparison Experiment

**Figure 9:**
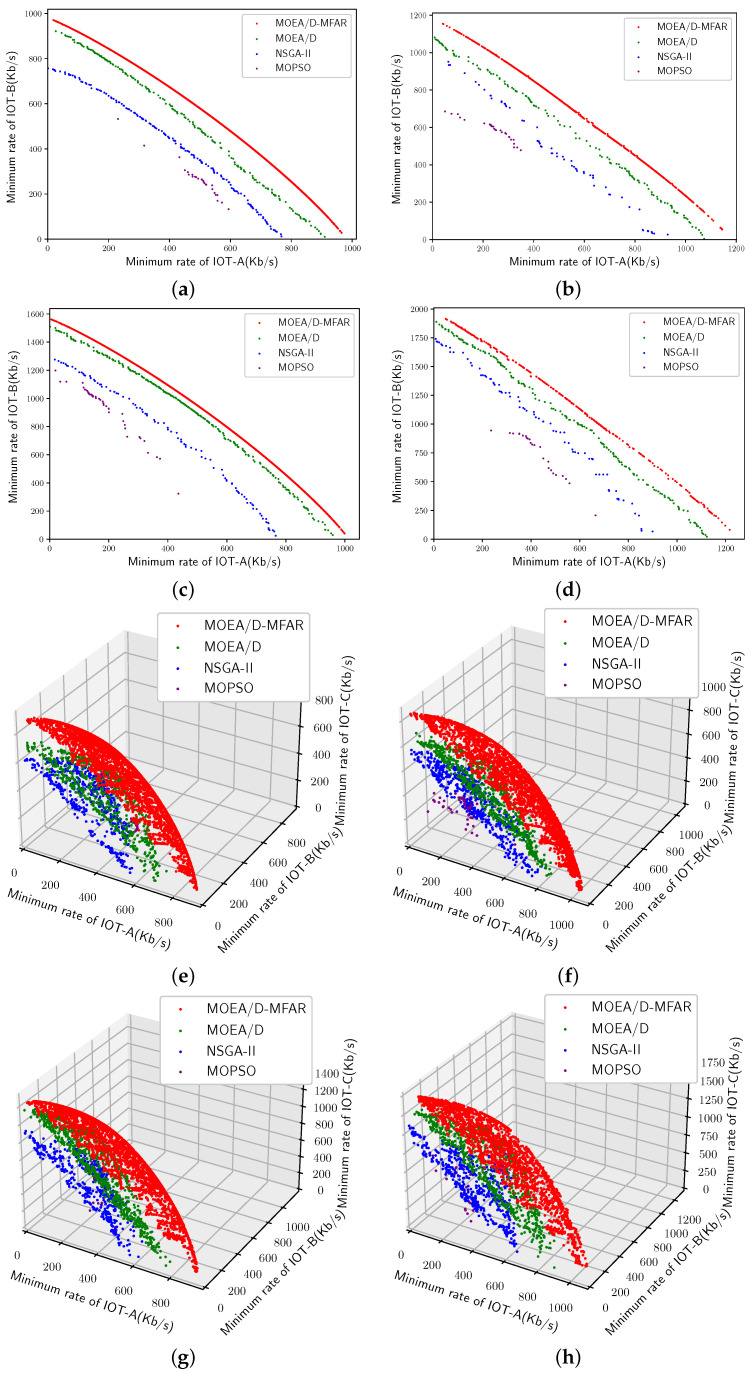
PFs of MOEA/D-MFAR, MOEA/D, NSGA-II, and MOPSO in eight scenarios. (**a**) Scenario 1. (**b**) Scenario 2. (**c**) Scenario 3. (**d**) Scenario 4. (**e**) Scenario 5. (**f**) Scenario 6. (**g**) Scenario 7. (**h**) Scenario 8.

This section compares four distinct algorithms’ performance across eight different scene settings. The algorithms under consideration are MOEA/D-MFAR, MOEA/D, NSGA-II, and MOPSO. To ensure a fair comparison, algorithm parameters such as population size, number of iterations, etc., are uniformly configured. Initially, the PFs under the eight scenarios are illustrated in Figure 9. Red scatter points represent MOEA/D-MFAR results, green scatter points denote MOEA/D, blue scatter points indicate NSGA-II outcomes, and purple scatter points signify MOPSO results. Evidently, regardless of the scenarios, MOEA/D-MFAR consistently outperforms other algorithms in obtaining superior PFs. Intuitively, MOEA/D-MFAR exhibits the best domination ability, followed by MOEA/D, NSGA-II, and MOPSO. Under the dual-objective optimization problems (refer to Figure 8a–d), MOEA/D-MFAR yields elongated PFs, while under the three-objective optimization problems (refer to Figure 8e–h), it manifests complete surfaces. This underscores the exceptional search capability of MOEA/D-MFAR within the target space. For a more objective analysis of algorithm performance, Table 5 displays the final HV values of PFs and the number of feasible solutions. The best values are bolded. On one hand, MOEA/D-MFAR exhibits an absolute advantage in HV value, indicating superior results in addressing this type of multi-objective optimization problem. This outcome is attributed to a more rational resource allocation. On the other hand, MOEA/D-MFAR attains the highest number of feasible solutions, showcasing its ability to address a broader range of dynamic demand scenarios.

**Figure 10:**
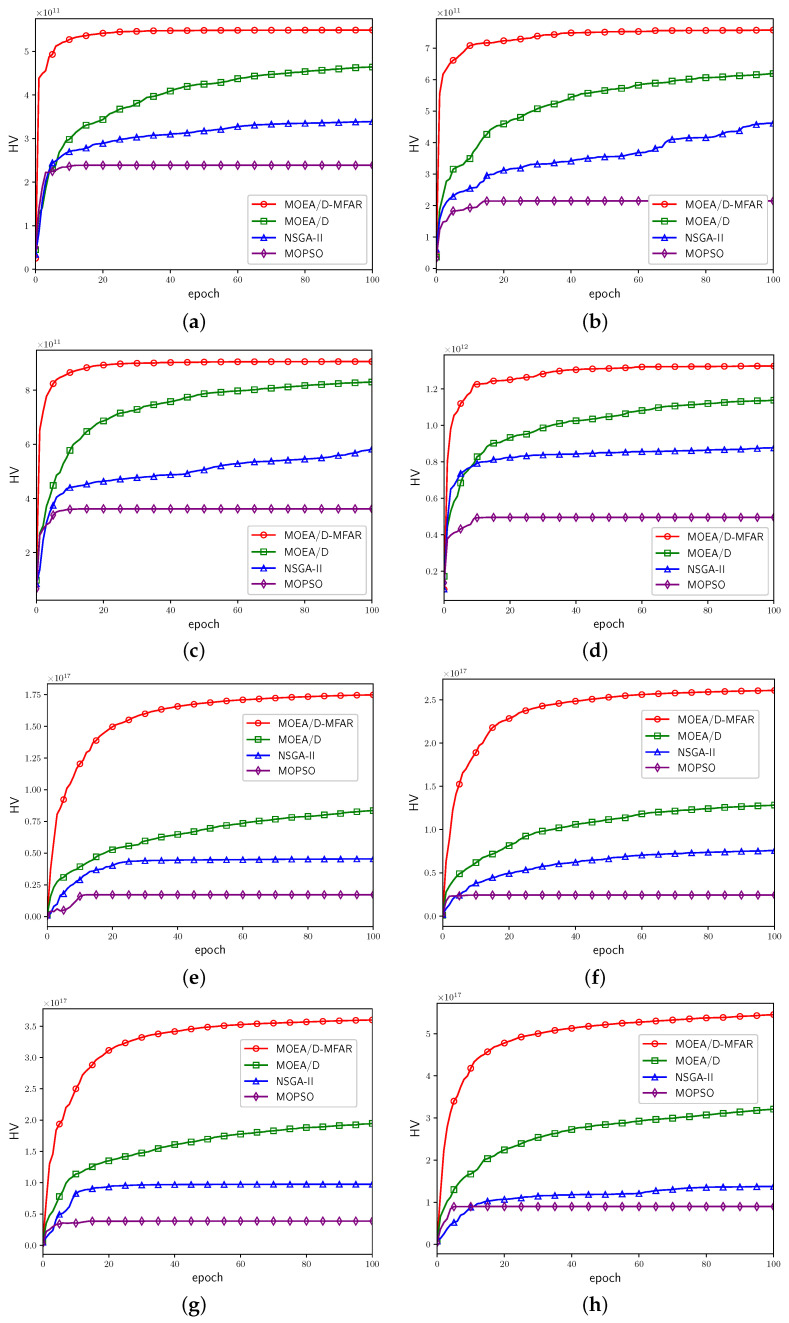
HV curves of MOEA/D-MFAR, MOEA/D, NSGA-II, and MOPSO in eight scenarios. (**a**) Scenario 1. (**b**) Scenario 2. (**c**) Scenario 3. (**d**) Scenario 4. (**e**) Scenario 5. (**f**) Scenario 6. (**g**) Scenario 7. (**h**) Scenario 8.

Moreover, to monitor the convergence process of different algorithms, the HV values for each epoch of each algorithm in various scenarios are recorded and depicted in Figure 10. Across all scenarios, the HV curves corresponding to MOEA/D-MFAR consistently reach the highest values, followed by MOEA/D, NSGA-II, and MOPSO. Each HV curve corresponding to MOEA/D exhibits a continuous upward trend, indicating that it has not yet converged throughout the optimization process. Conversely, the HV curves for NSGA-II and MOPSO display initial upward trends in the early epochs, but often experience slow or plateaued growth thereafter. This suggests that benchmark algorithms lack robust solution search capabilities for this type of problem, making them susceptible to local optima and resulting in slow convergence. Additionally, it is noteworthy that problems with varying numbers of optimization objectives exhibit different complexities. For the curves corresponding to MOEA/D-MFAR, it is apparent that convergence speed in the three-objective optimization problem (Figure 9e–h) is relatively slower than in the two-objective optimization problem (Figure 9a–d). In the multi-UAV scenario, convergence speed (Figure 9a,c,e,g) is slower compared to the single-UAV scenarios (Figure 9b,d,f,h).

Similarly, to assess the fairness of resource allocation among IoT devices of the same type, the ASDR values corresponding to the four algorithms are calculated and presented in Table 6. The best values are bolded. In relatively straightforward scenarios (such as scenarios 1, 3, 5, and 7), ASDR values are nearly equal to 0, signifying nearly identical channel capacity among devices. Thus, MOEA/D-MFAR demonstrates superior performance in minimizing channel rate differences among devices within similar IoT devices.

In summary, the superiority and robustness of MOEA/D-MFAR have been unequivocally demonstrated, surpassing the comparison algorithms MOEA/D, NSGA-II, and MOPSO. This achievement can be attributed to several key factors that set MOEA/D-MFAR apart from the comparison algorithms. MOEA/D-MFAR is distinguished by its incorporation of equitable resource allocation knowledge, a feature not present in conventional multi-objective optimization methodologies. This algorithmic approach allocates resources across different classes, followed by a nuanced distribution within each class, thereby enhancing the directness of the search process. Consequently, MOEA/D-MFAR achieves a higher efficiency in identifying feasible solutions compared to general multi-objective optimization algorithms. Furthermore, the decay rate mechanism within MOEA/D-MFAR is pivotal in mitigating oscillations around the optimal solution during the latter stages of the optimization process. This mechanism accelerates the convergence rate and facilitates a more refined resource allocation. As a result, it minimizes the variance in channel capacity among devices of the same category, thereby promoting a more nuanced and equitable distribution of resources across the system.

While MOEA/D-MFAR demonstrates significant advantages, it does have limitations. It necessitates the specification of numerous parameters, including mutation probabilities $\left(PB_{um},PB_{bdm},PB_{cm}\right)$ and scaling coefficients $\left(c_{1}∼c_{4}\right)$, in addition to those required by the MOEA/D framework. Moreover, its application is primarily tailored to scenarios that emphasize fairness in resource allocation, which may restrict its generalizability to other optimization contexts.

### 6.4. Sensitivity Analysis

It is necessary to conduct sensitivity analyses on the number of IoT devices and the number of UAVs, because the quantities of IoT devices and UAVs are very likely to vary in different real-world scenarios. In this way, the robustness of the model can be evaluated.

For the sensitivity analysis of the number of IoT devices, we constructed two scenarios, namely scenario A and scenario B. In these two scenarios, the numbers of IoT device types are 2 and 3, respectively, the population sizes are 100 and 120, respectively, and the number of algorithm iterations is 50 for both. Moreover, the distribution of ground devices is sparse, and other parameter settings are the same as those in Table 4. Similarly, we used the ADSR indicator for evaluation to observe the fairness of resource allocation. As the number of ground IoT devices increases, the ADSR indicators in scenario A and scenario B are presented in Table 7. It can be observed that as the number of devices increases, the ADSR shows a fluctuating and slowly rising trend. In scenario A, when the number of each type of ground IoT device reaches 1000, the ADSR is 2.28121 kb/s (bolded in the table), which is 2.08684 kb/s (bolded in the table) in scenario B. In the case where the number of each terminal is 1000, the ADSR is still within an acceptable range, which to some extent indicates that resources can still be allocated fairly, proving that the algorithm is also effective in the case of a large number of ground IoT devices.

Furthermore, for the sensitivity analysis of the number of UAVs, we set up scenario C and scenario D. In scenario C and scenario D, the types of IoT devices are 2 and 3, respectively, and the number of each type of device is 30. The devices are sparsely distributed, and other parameters are the same as those in Table 4. Similarly, we observed the changes in ASDR under different settings of the number of drones. The changes in ASDR in scenario C and scenario D are shown in Table 8. It can be found that whether in scenario C or scenario D, as the number of drones increases, the ASDR shows a fluctuating and slowly rising trend. This is partly because the resource allocation of UAVs involves discrete resource variables, that is, the association between IoT devices and UAVs, which will increase the difficulty of fair resource allocation. When the number of drones reaches 25, the ASDR in scenario C and scenario D reaches 2.30610 kb/s and 9.56433 kb/s, respectively (bolded in the table), which are also relatively small. Therefore, the algorithm maintains a certain level of effectiveness even in scenarios with a large number of UAVs.

Hence, it is observable that an increase in the number of IoT devices or UAVs leads to a heightened complexity in the resolution of the algorithm. This is accompanied by a trend of fluctuation and a slight yet acceptable increment in the ADSR. Therefore, the maximum number of devices and UAVs that the algorithm can handle can be limited based on the minimum requirements for the ADSR.

### 6.5. Scenario with a Large Number of Devices

In scenarios involving more than three categories of IoT devices, the PFs may not be easily demonstrated; yet, it is still crucial to monitor the performance of algorithms in environments with a multitude of device types. Consequently, we constructed a scenario comprising ten distinct categories of IoT devices, with each category containing ten devices, totaling one hundred IoT devices, and one UAV. The population size was set to 120, with all other settings consistent with Table 4. We listed the final ADSR for algorithm iterations of 10, 20, 30, 40, and 50 in Table 9. It was observed that as the number of iterations increased, the number of feasible solutions also increased, while the ADSR decreased. When the iteration count reaches 50, the ADSR is 2.54977 kb/s. Comparing this with Table 7, where each category of IoT devices had ten units, the ADSR is 0.03453 kb/s in scenario A (with two device types) and 1.47435 kb/s in scenario B (with three device types). As the number of device types increases, the ADSR experiences a modest increase, which can be attributed to the increased complexity of the algorithm. However, this increase remains within an acceptable range.

## 7. Conclusions

In conclusion, this study introduced and addressed the challenge of fair resource allocation in a diverse IoT device environment within the SAGIN framework. The problem was formulated as a multi-objective optimization problem, specifically aiming to maximize the minimum channel capacity for each category of IoT devices. The proposed MOEA/D-MFAR algorithm, incorporating a decay rate mechanism, showcased superior performance and convergence over three traditional algorithms. The decay rate mechanism significantly increased the number of feasible solutions, facilitating convergence, as verified through testing. Through extensive evaluation in various scenarios, MOEA/D-MFAR demonstrated its ability to generate a greater number of superior feasible solutions, thereby enhancing the minimum channel capacity for each type of IoT device while ensuring equitable resource distribution. The abundance of pre-provided feasible solutions enhances the adaptability to scenarios characterized by dynamic and evolving requirements. This research adeptly resolves resource allocation equity in static ground equipment scenarios for applications like fixed-point oceanic exploration and wilderness monitoring. However, its applicability to highly mobile equipment scenarios is not addressed. In the future, we will explore more complex scenarios that include more resource types and mobile terminals, and seek efficient solutions.

## Figures and Tables

**Table 1:** Comparison of related work.

Related Work	Scenario	Method	Objectives	Drawbacks
Ref. [26]	The UAV-LEO integrated data collection scenario in the B5G IoRT networks.	Successive convex approximation and block coordinate descent techniques.	The weighted function of the total data uploaded by UAVs, energy consumption, and the minimum channel capacity among IoT devices.	1. Ignoring the differences in device requirements. 2. Lacking the ability to quickly respond to changes in demand.
Ref. [27]	HAP-reserved communications in SAGIN.	Decompose into two sub-problems and solve them using convex optimization and linear programming, respectively.	Maximize total system throughput.	1. Ignoring the differences in device requirements. 2. Lacking the ability to quickly respond to changes in demand.
Ref. [28]	Integrated satellite–airborne–terrestrial network for downlink communication.	Fix HAP position, solve binary linear optimization with Taylor expansion, then optimize HAP location via recursive contraction and relocation.	Maximize the downlink channel capacity for users.	1. Ignoring the differences in device requirements. 2. Lacking the ability to quickly respond to changes in demand. 3. Ignoring fairness among similar users.
Ref. [29]	UAV-based SAG-IoRT uplink channel transmission.	Joint iterative optimization using LP, variable transformation, continuous convex, and block coordinate descent.	Maximize the system’s channel capacity.	1. Ignoring the differences in device requirements. 2. Lacking the ability to quickly respond to changes in demand. 3. Ignoring fairness among similar devices.
Ref. [30]	UAVs provide sustainable communication services for ground devices.	Low-complexity iterative suboptimal algorithm based on continuous convex approximation.	Maximize the system’s channel capacity.	1. Ignoring the differences in device requirements. 2. Lacking the ability to quickly respond to changes in demand. 3. Ignoring fairness among similar devices.
Ref. [31]	UAVs provide sustainable communication services for ground devices.	Iterative joint optimization method based on continuous convex optimization and block coordinate descent algorithm.	Maximize the minimum throughput of users.	1. Ignoring the differences in device requirements. 2. Lacking the ability to quickly respond to changes in demand.
Ref. [32]	SAGIN base on airship for ground signal coverage.	Multi-objective optimization algorithm-based MOEA/D.	Maximize coverage range and network speed.	1. Ignoring fairness among similar users.

**Table 2:** Notation summary.

Symbol	Description
$T,I_{t}$	The number of types of IoT devices, the *t*-th category of IoT devices.
$N_{t},N^{I},N^{U}$	The number of the *t*-th category of IoT devices, the total number of IoT devices, the number of UAVs.
$\left(x_{n}^{t},y_{n}^{t}\right)$ $\left(x_{n}^{U},y_{n}^{U},h^{U}\right)$	Coordinate of the *n*-th device in $I_{t}$, coordinate of the *n*-th UAV.
*B*	The total available bandwidth of the system.
$j,k^{t},K$	The set of bandwidth ratios occupied by different types of IoT devices, the set of the bandwidth ratios occupied by each device in the t-th category of IoT devices, the set of $k^{t}$ for each category of IoT devices.
$j_{t},k_{n}^{t}$	The proportion of total bandwidth occupied by the *t*-th category of IoT devices, the proportion of bandwidth occupied by the *n*-th device in the *t*-th category of IoT devices.
$a_{t},A$	The set of UAV serial numbers selected by each device in the *t*-th category of IoT devices, the set of $a_{t}$ for each category of IoT devices.
$\alpha_{tn},C_{tn}^{U}$	The UAV sequence number selected by the *n*-th device in the *t*-th-category IoT devices, the number of IoT devices associated with the UAV selected by the *n*-th device in the *t*-th-category IoT devices.
$P^{I},P^{U}$	Signal transmission power of IoT devices, total forwarding power per UAV.
$\left(x^{L},y^{L},h^{L}\right)$	The three-dimensional coordinate of an LEO satellite.
$h^{U},h^{L}$	The fixed height at which the drone flies, the altitude of the LEO satellite.
$g_{0}$	The channel gain per unit distance.
$g_{n}^{I_{t}\to U_{\alpha_{tn}}}$ $g_{n}^{U_{\alpha_{tn}}\to L}$	The channel gain between the *n*-th device in $I_{t}$ and the selected UAV, the channel gain between the UAV selected by the *n*-th device in $I_{t}$ and LEO satellite.
$\gamma_{n}^{I_{t}\to U_{\alpha_{tn}}}$ $\gamma_{n}^{U_{\alpha_{tn}}\to L}$	The signal-to-noise ratio between the *n*-th device in $I_{t}$ and the selected UAV, the signal-to-noise ratio between the UAV selected by the *n*-th device in $I_{t}$ and LEO satellite.
$N_{0}$	The power spectral density of additive white Gaussian noise.
$B_{n}^{t},P_{n}^{t}$	The bandwidth occupied by the *n*-th device in $I_{t}$, the forwarding power obtained by the *n*-th device in $I_{t}$ from the selected UAV.
$R_{n}^{t}$	The channel rate of the *n*-th device in $I_{t}$ forwarded to the LEO satellite via a UAV.

**Table 3:** Details of the eight scenarios.

Scenario	Number of Types of IoT Devices	Number of UAV	Distribution Characteristics of IoT Devices	The Respective Quantities of Different Types of IoT Devices
1	2	1	sparse	10, 10
2	2	3	sparse	10, 10
3	2	1	dense	10, 6
4	2	3	dense	10, 6
5	3	1	sparse	10, 10, 10
6	3	3	sparse	10, 10, 10
7	3	1	dense	10, 8, 6
8	3	3	dense	10, 8, 6

**Table 4:** Simulation parameters.

Parameter	Value
Height of UAV and LEO satellite	100 m, 1000 km
System bandwidth *B*	1 MHz
Channel gain per unit distance $g_{0}$	$1.42\times 10^{-4}$
Total transmission power of UAV $P^{U}$	50 W
Transmission power of IoT device $P^{I}$	1 W
Noise power spectral density $N_{0}$	−174 dBm/Hz
Population size *M*	100 for scenarios 1,2,3,4
	120 for scenarios 5,6,7,8
Iteration steps *E*	100
Neighborhood Size *S*	5
$c_{1},c_{2},c_{3},c_{4}$	0.1, 0.5, 0.5, 0.5
Base of decay rate *b*	0.8 for scenarios 1,3,5,7
	0.7 for scenarios 2,4,6,8
$PB_{um},PB_{bdm},PB_{cm}$	0.5, 0.5, 0.1

**Table 5:** HV and number of feasible solutions.

Algorithm	MOEA/D-MFAR	MOEA/D-MFAR-ndr	MOEA/D	NSGA-II	MOPSO	MOEA/D-MFAR	MOEA/D-MFAR-ndr	MOEA/D	NSGA-II	MOPSO
Scenario	Scenario 1					Scenario 2				
HV	$5.49\times 10^{11}$	$5.19\times 10^{11}$	$4.64\times 10^{11}$	$3.39\times 10^{11}$	$2.39\times 10^{11}$	$7.57\times 10^{11}$	$6.71\times 10^{11}$	$6.19\times 10^{11}$	$4.61\times 10^{11}$	$2.15\times 10^{11}$
Number of solution	**4290**	113	197	165	26	**977**	102	134	64	39
Scenario	Scenario 3					Scenario 4				
HV	$9.06\times 10^{11}$	$8.4\times 10^{11}$	$8.3\times 10^{11}$	$5.82\times 10^{11}$	$3.62\times 10^{11}$	$1.33\times 10^{12}$	$1.13\times 10^{12}$	$1.13\times 10^{12}$	$8.76\times 10^{11}$	$4.95\times 10^{11}$
Number of solution	**4620**	106	252	97	44	**346**	80	166	77	24
Scenario	Scenario 5					Scenario 6				
HV	$1.75\times 10^{17}$	$1.13\times 10^{17}$	$8.36\times 10^{16}$	$4.54\times 10^{16}$	$1.72\times 10^{16}$	$2.61\times 10^{17}$	$1.77\times 10^{17}$	$1.28\times 10^{17}$	$7.57\times 10^{16}$	$2.43\times 10^{16}$
Number of solution	**3639**	383	679	642	113	**3908**	380	729	661	83
Scenario	Scenario 7					Scenario 8				
HV	$3.6\times 10^{17}$	$2.42\times 10^{17}$	$1.95\times 10^{17}$	$9.76\times 10^{16}$	$3.86\times 10^{16}$	$5.45\times 10^{17}$	$3.62\times 10^{17}$	$3.21\times 10^{17}$	$1.37\times 10^{17}$	$8.98\times 10^{16}$
Number of solution	**3612**	370	888	509	56	**2731**	431	747	584	23

**Table 6:** ASDR of different algorithms.

ASDR (kb/s)	Scenario 1	Scenario 2	Scenario 3	Scenario 4	Scenario 5	Scenario 6	Scenario 7	Scenario 8
MOEA/D-MFAR	**0.00472**	**4.88970**	**0.00638**	**6.06020**	**0.78157**	**3.81755**	**0.55242**	**4.39425**
MOEA/D-MFAR-ndr	6.71880	21.21205	15.34665	24.34125	24.09956	32.22496	32.87763	53.12022
MOEA/D	37.91355	48.36469	27.09743	45.89225	63.04678	74.17693	54.01430	78.72201
NSGA-II	36.54003	105.26025	33.97831	89.76500	94.81778	112.85205	114.71659	154.59454
MOPSO	164.07773	140.23824	169.52251	189.42786	89.40870	163.86959	148.69868	173.26047

**Table 7:** Sensitivity analysis of number of IoT devices.

Number of IoT Devices per Category	10	30	50	70	100	200	300	400	1000
ADSR(kb/s) scenario A	0.03453	0.00859	0.00519	0.00388	0.00318	0.00199	0.73537	1.86991	**2.28121**
ADSR(kb/s) scenario B	1.47435	3.94150	1.15200	1.44833	3.28922	4.67727	5.10086	3.94150	**2.08684**

**Table 8:** Sensitivity analysis of number of UAVs.

Number of UAV	1	2	3	5	10	15	20	25
ADSR(kb/s) scenario C	0.00859	2.29062	1.74026	0.96567	1.63528	2.24172	2.57945	**2.30610**
ADSR(kb/s) scenario D	3.94150	3.35178	1.67385	1.82613	4.42636	6.20919	6.19233	**9.56433**

**Table 9:** The variation in ADSR with the number of iterations in a scenario with 10 types of IoT devices.

Iterations	10	20	30	40	50
ADSR(kb/s)	13.81754	7.44094	5.24779	4.38385	2.54977
Number of solutions	915	1811	2876	4021	5044

## Data Availability

Data are contained within the article.
